# *OIP5-AS1* specifies p53-driven POX transcription regulated by TRPC6 in glioma

**DOI:** 10.1093/jmcb/mjab001

**Published:** 2021-01-28

**Authors:** Wei Shao, Zhen-Yu Hao, Yi-Fei Chen, Jun Du, Qian He, Liang-Liang Ren, Yan Gao, Nan Song, Yan Song, Hua He, Yi-Zheng Wang

**Affiliations:** 1 The Brain Science Center, Beijing Institute of Basic Medical Sciences, Beijing 100850, China; 2 Department of Neurosurgery, Changzheng Hospital, Second Military Medical University, Shanghai 200003, China

**Keywords:** DNA‒RNA hybrid, glioma, lncRNA, p53, transcription

## Abstract

Transcription factors (TFs) control an array of expressed genes. However, the specifics of how a gene is expressed in time and space as controlled by a TF remain largely unknown. Here, in TRPC6-regulated proline oxidase 1 (POX) transcription in human glioma, we report that *OIP5-AS1*, a long noncoding RNA, determines the specificity of p53-driven POX expression. The *OIP5-AS1*/p53 complex via its 24 nucleotides binds to the POX promoter and is necessary for POX expression but not for p21 transcription. An O-site in the POX promoter to which *OIP5-AS1* binds was identified that is required for *OIP5-AS1*/p53 binding and POX transcription. Blocking *OIP5-AS1* binding to the O-site inhibits POX transcription and promotes glioma development. Thus, the *OIP5-AS1*/O-site module decides p53-controlled POX expression as regulated by TRPC6 and affects glioma development.

## Introduction

Gene expression is a fundamental biological event linked to cell proliferation, differentiation, and survival. Transcription factors (TFs) play a decisive role in promoting gene expression at the right time and in the right place. They bind to specific sequences of genomic DNA via their DNA-binding domains (DBDs) to regulate expression (Lambert et al., 2018). For example, p53, a TF and a well-known tumor suppressor, is crucial for the cell cycle (Shaw, 1996) and the expression of a spectrum of genes, including MDM2, p21, BAX, PTEN, and proline oxidase 1 (POX) (Polyak et al., 1997; May and May, 1999). It regulates gene expression by binding to the target gene’s promoter DNA sequence. The p53-binding site in the genomes of mammals is comprised of a half-site 5ʹ-RRRCWWGYYY-3ʹ followed by a spacer, which is then followed by a second half-site RRRCWWGYY sequence (Ma et al., 2007; Riley et al., 2008). But when p53 is activated, not all p53 target genes are expressed at the same time (Beckerman and Prives, 2010). How p53 controls gene expression specifically by a consensus binding site remains largely unknown.

Long noncoding RNAs (lncRNAs), transcripts of >200 nucleotides that are not translated into proteins, have emerged as key regulators of the transcriptional network in development and disease (Fatica and Bozzoni, 2014). It has been widely reported that lncRNAs can exert diverse functions by binding to protein, RNA, and DNA, including transcriptional regulation in cis or trans and organization of nuclear domains (Fatica and Bozzoni, 2014; Kopp and Mendell, 2018). *OIP5-AS1*, a lncRNA first identified in zebrafish, plays an important role in embryogenesis. Knocking down *OIP5-AS1* in zebrafish results in small heads and eyes, as well as short, curly tails of the morphants (Ulitsky et al., 2011). In HeLa cells, *OIP5-AS1* prevents HuR binding to its target mRNA to further inhibit cell proliferation (Kim et al., 2016).

In human glioma, the expression of transient receptor potential canonical 6 (TRPC6), known as a channel protein (Nishida et al., 2015), is upregulated and the inhibition of its expression or activities by RNAi or the dominant-negative form of TRPC6 (DNC6) suppresses glioma cell proliferation and glioma development (Ding et al., 2010). However, it remains unclear how TRPC6 regulates glioma development. Proline dehydrogenase (PRODH)/POX is encoded by p53-induced gene 6 (PIG-6), and POX can also initiate apoptosis in colorectal cancer cells (Liu et al., 2005; Phang, 2019).

Here, we report that *OIP5-AS1* specifically mediates p53-driven POX expression, which is crucial for the effects of TRPC6 on human glioma development. In particular, we found that *OIP5-AS1* mediates p53 binding to the POX promoter by 24 nucleotides (24-nt motif) that pair with the O-site in the POX promoter. Blocking *OIP5-AS1* binding to the O-site by a decoy or CRISPR/Cas9 inhibits POX expression and promotes glioma development.

## Results

### POX expression is specifically regulated by TRPC6 in human glioma

**Figure 1:**
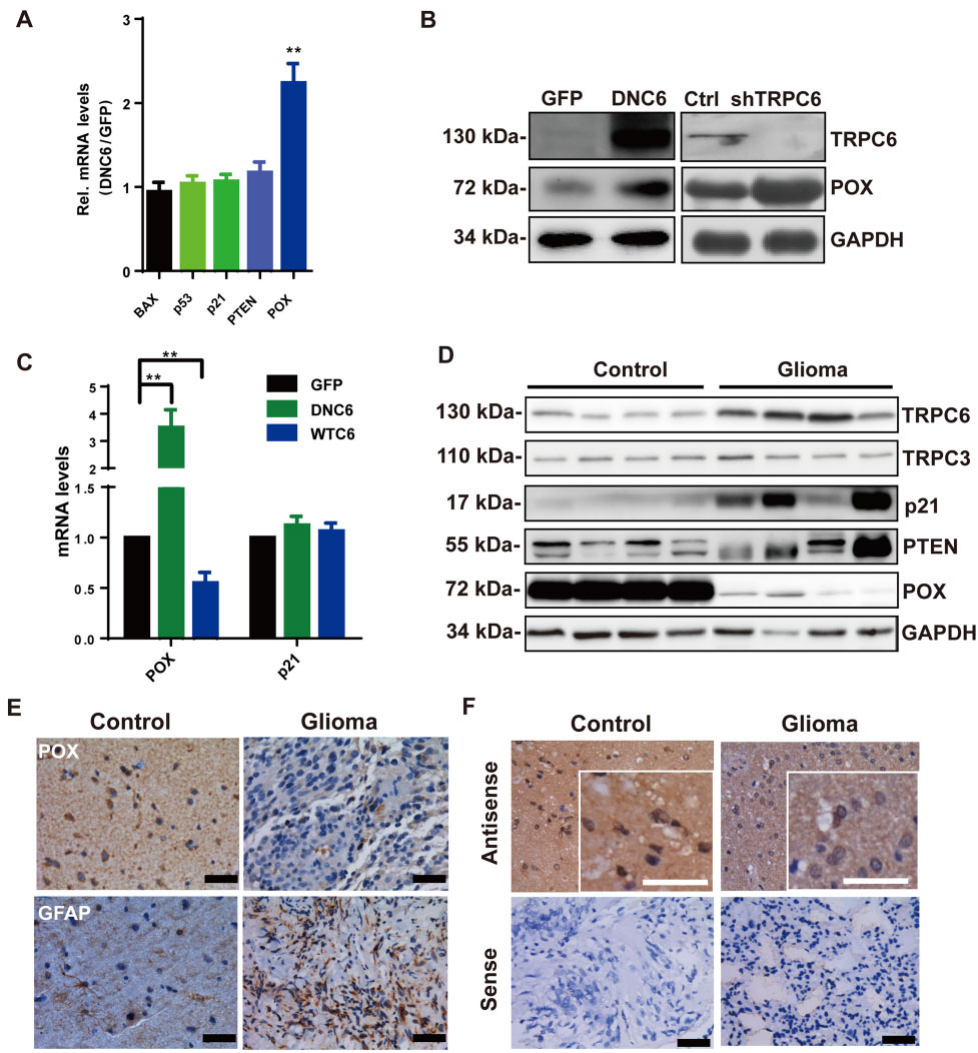
Specific POX expression regulated by TRPC6 in human glioma. (**A**) qPCR analysis of the indicated mRNA levels in LN229 cells. (**B**) Immunoblotting of total lysates of LN229 cells transfected with GFP and DNC6 (left panel) or control shRNA and shTRPC6 (right panel) with the indicated antibodies. (**C**) qPCR analysis of POX and p21 mRNA levels in LN229 cells transfected with GFP, DNC6, or WT-TRPC6 (WTC6). (**D**) Immunoblotting of total lysates from 40 glioma and 39 human normal brain (control) tissues (four samples are shown) probed with the indicated antibodies. GAPDH as loading controls. (**E**) Immunohistological staining in glioma and control human brain tissues with hematoxylin, GFAP, and POX antibodies. Scale bar, 50 µm. (**F**) POX mRNA expression in glioma and control tissues detected by *in situ* hybridization. Antisense probe: POX mRNA; sense probe: negative control. Scale bar, 50 µm (black) or 10 µm (white). Unless stated, data are mean ± SEM of at least three independent experiments in triplicate, *t*-test, **P < 0.01.

TRPC6 is a channel protein important for cancer devolvement and human glioma cell proliferation (El Boustany et al., 2008; Cai et al., 2009; Chigurupati et al., 2010). We found that both the mRNA and protein levels of POX in LN229 human glioma cells were upregulated (Figure 1A and B; Supplementary Figure S1A) when TRPC6 activity or expression was inhibited by DNC6 (Hofmann et al., 2002) or by RNAi. In contrast, the expression levels of the tumor suppressor genes BAX, p53, p21, and PTEN (Chen et al., 2012) were not changed (Figure 1A). Consistently, expressing TRPC6 greatly suppressed POX but not p21 (Figure 1C). Next, in ∼75% of >40 human glioma samples, the protein expression level of TRPC6 or p21 was ∼3.3- or 3.5-fold higher whereas that of POX or PTEN was ∼6.4- or 1.4-fold lower than in normal tissues (Figure 1D;Supplementary Figure S1B). In contrast, there was no difference in the mean levels of TRPC3 expression between the glioma and normal tissues (Figure 1D;Supplementary Figure S1B). Further analysis of the data obtained from The Cancer Genome Atlas (TCGA) revealed that there was an inverse correlation between the mRNA expression of TRPC6 and that of POX. However, we could not find a correlation between the mRNA expression of TRPC6 and that of p53 or PTEN (Supplementary Figure S2A). Furthermore, immunohistology of tissues using an anti-POX antibody showed that normal glial (para-tumor) tissues that were GFAP-positive exhibited strong immunostaining, while few signals were noted in the glioma samples (Figure 1E;Supplementary Figure S2B). Similarly, the POX mRNA level in glioma tissues was markedly reduced (Supplementary Figure S2C). *In situ* hybridization showed that the signal of POX mRNA was decreased in grade 4 glioma tissues compared to nontumor tissues (Figure 1F). Thus, TRPC6 specifically regulates POX expression in human glioma cells, and their expression levels are inversely correlated in human glioma tissues.

### The dependence of TRPC6-regulated POX expression on p53

**Figure 2:**
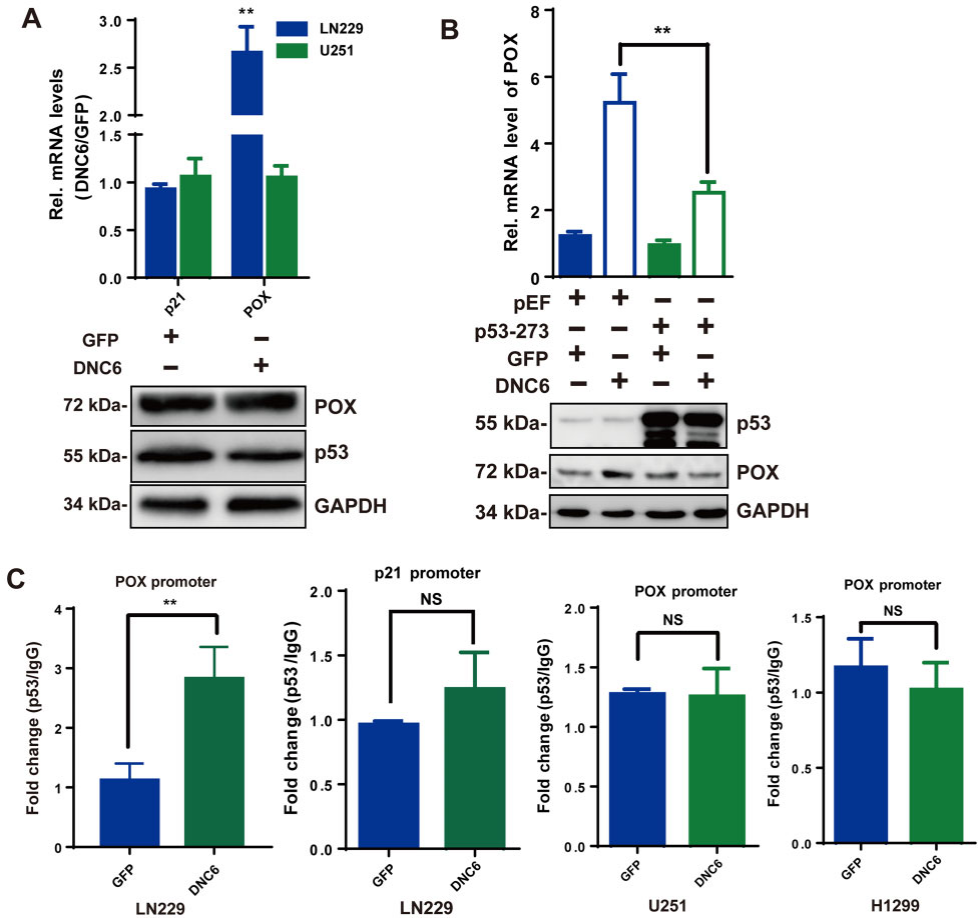
Dependence of TRPC6-regulated POX expression on p53 binding with the POX promoter. (**A**) POX and p21 mRNA levels (upper) and POX and p53 protein levels (lower) in U251 cells transfected with GFP or DNC6. (**B**) POX mRNA levels (upper) and POX and p53 protein levels (lower) in LN229 cells transfected with p53 (R273H) or DNC6. (**C**) ChIP analysis with p53 or IgG antibodies in LN229, U251, or H1299 cells. Unless stated, data are mean ± SEM of at least three independent experiments in triplicate, *t*-test, **P < 0.01, NS indicates no significance.

We then asked how TRPC6 controls POX but not BAX, p21, or PTEN expression in human glioma cells. The upregulation of POX expression at both mRNA and protein levels was not observed in U251 cells, a human glioma cell line that harbors a mutated form of p53 (R273H) (Figure 2A), or H1299 cells, a human lung carcinoma cell line that lacks p53 (Supplementary Figure S2D; Giaccone et al., 1992). Moreover, overexpressing p53 (R273H) in LN229 cells suppressed the DNC6-induced POX upregulation (Figure 2B). Therefore, TRPC6 might specifically regulate POX expression via p53. This assumption was further supported by the finding that p53 or POL II association with the POX promoter was greatly enhanced by DNC6, while p53 association with the p21 promoter was not affected in chromatin immunoprecipitation (ChIP) experiments (Figure 2C;Supplementary Figure S2E). In U251 and H1299 cells, inhibition of TRPC6 did not induce p53 association with the POX promoter (Figure 2C). Collectively, these results suggest that TRPC6 regulates p53 association specifically with the POX promoter to control its expression in glioma cells.

### The OIP5-AS1/p53 complex is important for TRPC6-regulated POX expression

**Figure 3:**
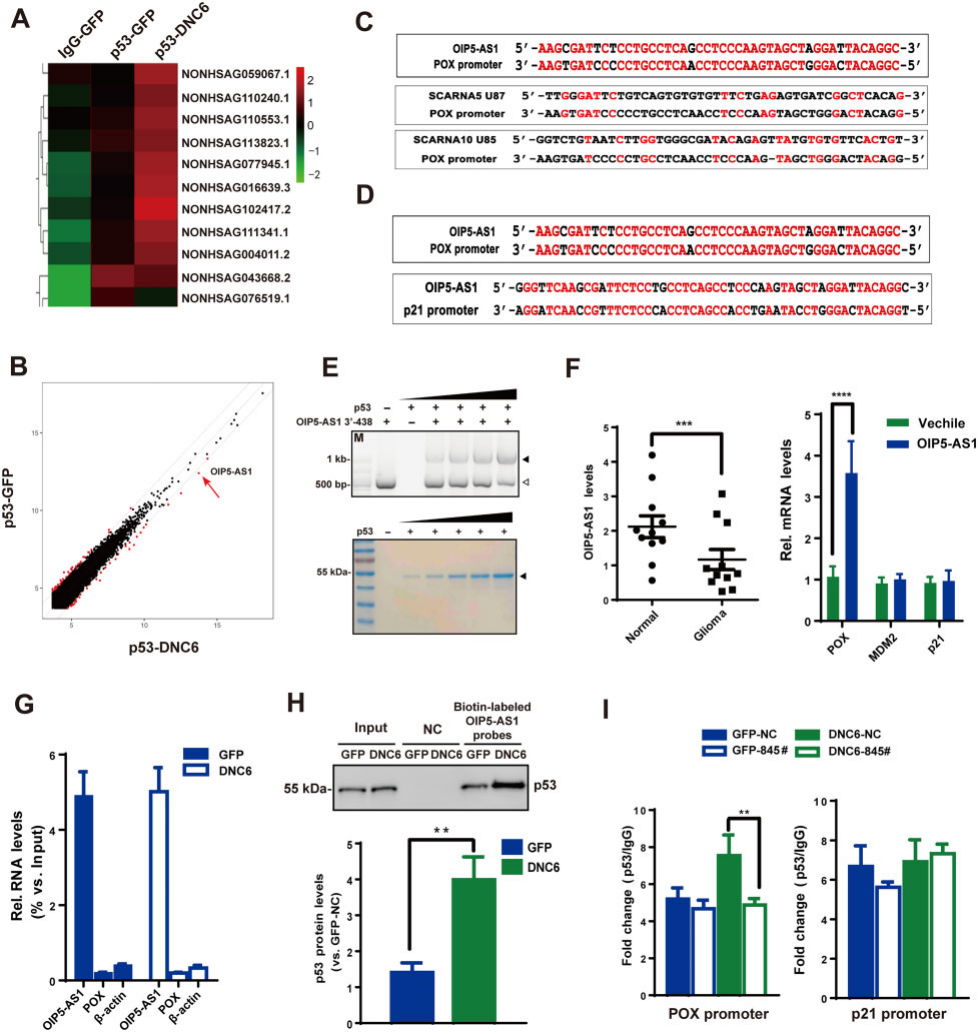
*OIP5-AS1*/p53 complex is important for TRPC6-regulated POX expression. (**A** and **B**) Heatmap and scatter plot of RIP–seq analysis of LN229 cells transfected with GFP or DNC6 using p53 or IgG antibodies. (**C**) BLAST analysis of the immunoprecipitated lncRNAs and the POX promoter. Letters in red indicate the complementary region. (**D**) BLAST analysis of *OIP5-AS1*, POX (region of 3000 nt upstream of transcription start site, upper panel), and p21 (region of 3000 nt upstream of transcription start site lower panel) promoter sequences. Letters in red indicate the complementary region. (**E**) Interaction of p53 with 3ʹ-438 assayed by EMSA. Upper: shift of 3ʹ-438 (white arrow) to the higher position (black arrow) in the presence of p53. Lower: Coomassie blue staining of p53 protein. (**F**) qPCR analysis of *OIP5-AS1* levels in glioma or normal brain tissues (left) and POX, MDM2, and p21 mRNA levels in LN229 cells transfected with *OIP5-AS1* for 48 h (right). β-actin as an endogenous control. (**G**) *OIP5-AS1* enrichment in ChIRP using 3ʹ-biotin-labelled *OIP5-AS1* probes and assayed by qPCR in LN229 cells transfected with GFP or DNC6. POX and β-actin mRNAs as controls. (**H**) Immunoblotting of lysates precipitated by *OIP5-AS1* probes in ChIRP assay using p53 antibody and quantification of p53 protein levels. Probes, a pool of 20 different 3ʹ-biotin-labelled probes covering the whole region of *OIP5-AS1*. NC, without probes. (**I**) ChIP analysis with p53 or IgG antibodies in LN229 cells transfected with GFP and DNC6 with or without 845#. NC, scramble single-strand RNA. 845#, the antisense oligo of *OIP5-AS1*. Unless stated, data are mean ± SEM of at least three independent experiments in triplicate, *t*-test, **P < 0.01, ***P < 0.001, ****P < 0.0001.

We next studied how inhibiting TRPC6 induces p53 association with the POX promoter but not with the p21 promoter. It has been noted that gene transcription can be affected by lncRNAs, molecules known to bind to protein and DNA (Ponting et al., 2009). Thus, we screened for lncRNAs precipitated by the antibody against p53 using RNA immunoprecipitation coupled with deep sequencing (RIP−seq). The RIP−seq from LN229 cells expressing DNC6 showed that 6 lncRNAs were enriched in the p53-precipitated complexes, including *OIP5-AS1* (NONHSAG016639.3), known as a tumor suppressor and important for development (Figure 3A and B; Ulitsky et al., 2011; Chiu et al., 2018; Kleaveland et al., 2018). The expression levels of these 6 lncRNAs in LN229 cells were then confirmed by quantitative real-time PCR (qPCR) analysis (Supplementary Figure S3A). Since it has been reported that some lncRNAs can recognize their targets in a manner dependent on base pairing (Ponting et al., 2009), we then aligned the 6 lncRNAs with the POX promoter and found that *OIP5-AS1* contains a 24-nt sequence well paired with a part of the POX promoter (Figure 3C;Supplementary Figure S3B). Moreover, the complementary 24-nt motif was found exclusively in the POX but not p21 or MDM2 promoter region (Figure 3D;Supplementary Figure S3C). Further RIP assays using three pairs of primers designed to amplify different exons of *OIP5-AS1* showed that *OIP5-AS1* was enriched by 7.5- to 11.0-fold (Supplementary Figure S3D). *OIP5-AS1* association with p53 was markedly increased when TRPC6 was inhibited, suggesting that the *OIP5-AS1* and p53 interaction was regulated by TRPC6 (Supplementary Figure S3D). Furthermore, in an electrophoretic mobility shift assay (EMSA), an *OIP5-AS1* 3ʹ-terminal 438-nt fragment (3ʹ-438) containing the 24-nt region was shifted to a higher position in the presence of recombinant p53, and this shift was p53 concentration-dependent (Figure 3E). Therefore, *OIP5-AS1* can directly bind with p53. Moreover, OIP5-AS1 expression was downregulated in glioma tissues, and its overexpression enhanced POX but not MDM2 or p21 expression in glioma cells (Figure 3F;Supplementary Figure S3E). Furthermore, overexpressing OIP5-AS1 in cells in which OIP5-AS1 was downregulated restored the upregulation of POX expression by DNC6 (Supplementary Figure S4A). We next performed the chromatin isolate by RNA precipitation (ChIRP) assay and found that *OIP5-AS1* probes with 3ʹ-biotin specifically enriched *OIP5-AS1* from 0.05% to 4.3% of the input (Figure 3G). Immunoblotting of ChIRP lysates showed that inhibition of TRPC6 greatly enhanced the association of p53 with *OIP5-AS1* in the cells (Figure 3H). Consistently, knockdown of *OIP5-AS1* (by 845#) (Lennox and Behlke, 2016) greatly suppressed p53 association with the POX promoter as assayed in ChIP experiments, whereas it did not affect p53 association with the p21 promoter (Figure 3I;Supplementary Figure S3F and G). Collectively, these results suggest that *OIP5-AS1* interacts with p53, and inhibition of TRPC6 enhances the interaction to increase POX expression.

### Direct binding of OIP5-AS1 to the POX promoter via its 24 nucleotides

**Figure 4:**
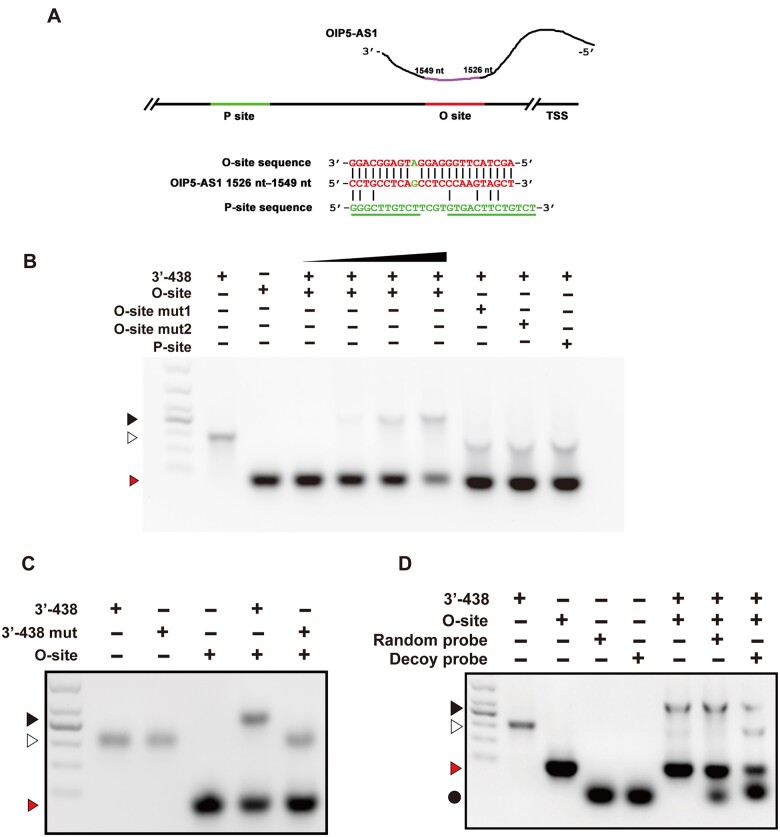
Direct binding of *OIP5-AS1* to the POX promoter via its 24 nucleotides. (**A**) Diagram of *OIP5-AS1* or p53 binding sites in POX promoter (Maxwell and Kochevar, 2008). Green: P-site (contains p53-binding consensus sequence); red: O-site. Letters in red indicate base pairing between the O-site and 1526‒1549 nt of *OIP5-AS1*. (**B**‒**D**) EMSA of 3ʹ-438 binding to O-site or P-site. (**B**) 3ʹ-438 binding to O-site (130 bp), its mutants (O-site mut1 or mut2), or P-site (130 bp). Red arrow: 130 bp containing O-site or its mutants; white arrow: 3ʹ-438; black arrow: RNA‒DNA (3ʹ-438:130 bp) hybrids. (**C**) 3ʹ-438 or its mutant (3ʹ-438 mut) binding to O-site. Red arrow: 130 bp containing O-site; white arrow: 3ʹ-438 or 3ʹ-438 mutant; black arrow: RNA‒DNA (3ʹ-438:130 bp) hybrids. (**D**) Blockage of 3ʹ-438 binding to O-site by the decoy probe. Circle: random or decoy probes; red arrow: 130 bp containing O-site; white arrow: 3ʹ-438; black arrow: RNA‒DNA (3ʹ-438:130 bp) hybrids.

Since *OIP5-AS1* contains a 24-nt sequence that is highly complementary with a part (defined as the O-site) of the POX promoter (Figure 4A), we thus examined whether *OIP5-AS1* directly binds to the POX promoter via the 24-nt region, which lacks a canonical p53-binding motif (Maxwell and Kochevar, 2008; Beckerman and Prives, 2010). In EMSA, 3ʹ-438, which contains the 24-nt region, was shifted to a higher position in the presence of a 130-bp fragment (containing the O-site) of the POX promoter, and this shift was 3ʹ-438 concentration-dependent (Figure 4B). The 3ʹ-438 shift was not observed when the 130-bp oligos were mutated at the O-site (mut1, mut2) or when a fragment containing the canonical p53-binding sequence (defined as the P-site, Figure 4A) of the POX promoter was utilized (Figure 4B). Reciprocally, the replacement in the 24-nt region of 3ʹ-438 blocked the 3ʹ-438 shift (Figure 4C). To confirm whether the hybrid formation of 3ʹ-438 and the 130-bp oligo was indeed dependent on the 24-nt region, we synthesized a 24-nt antisense DNA oligo (as a decoy probe) and examined its effects on the 3ʹ-438 shift. As shown in Figure 4D, the shift was noticeably suppressed by the decoy probe, but not by a random probe. These observations provided initial evidence to suggest that *OIP5-AS1* directly binds to the O-site in the POX promoter via its 24-nt region.

### Effects of OIP5-AS1/O-site module on p53 binding to the POX promoter

**Figure 5:**
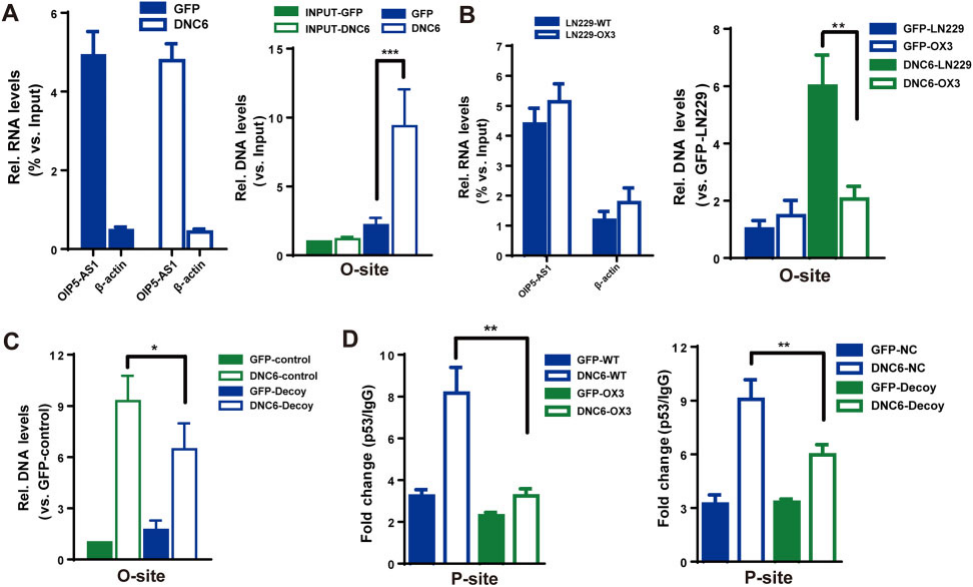
Effects of *OIP5-AS1*/O-site module on p53 association with the POX promoter. (**A**) qPCR analysis of *OIP5-AS1* enrichment by *OIP5-AS1* probes (left) and O-site levels (right) by ChIRP assay in LN229 cells transfected with GFP or DNC6. β-actin mRNA as a control. (**B**) qPCR analysis of *OIP5-AS1* (left) and O-site (right) levels by *OIP5-AS1* ChIRP assay in LN229 or OX3 (O-site mutant) cells transfected with GFP or DNC6. β-actin mRNA as a control. (**C**) qPCR analysis of O-site levels by *OIP5-AS1* ChIRP assay in LN229 cells transfected with GFP or DNC6 with or without the decoy. (**D**) ChIP analysis of P-site levels with p53 or IgG antibodies in LN229 (WT) or OX3 cells transfected with GFP or DNC6 (left) or in LN229 cells cotransfected with or without the decoy (right). NC, negative control with scramble decoy. Unless stated, data are mean ± SEM of at least three independent experiments in triplicate, *t*-test, *P < 0.05, **P < 0.01, ***P < 0.001.

To investigate whether *OIP5-AS1* could bind to the O-site in cells, we performed ChIRP assays and found that inhibition of TRPC6 greatly enhanced *OIP5-AS1* binding to the O-site in the POX promoter (Figure 5A). In comparison, such enhancement was not observed for the p21 promoter (Supplementary Figure S4B). Furthermore, we generated a knock-in LN229 cell line named OX3 in which the O-site was replaced by using a CRISPR/Cas9 approach (Supplementary Figure S4C). ChIRP assays showed that *OIP5-AS1* binding to the POX promoter was abolished in OX3 cells (Figure 5B). To minimize off-target effects caused by CRISPR/Cas9 and further confirm *OIP5-AS1* via the 24-nt region binding to the O-site in cells, we next designed a modified DNA decoy (*in vivo* morpholino) complementary to the 24-nt region that does not affect *OIP5-AS1* expression and degradation (Summerton, 1999). *OIP5-AS1* binding to the O-site in LN229 cells was greatly suppressed by the presence of the decoy (Figure 5C). Moreover, ChIP assays showed that p53 binding to the P-site in OX3 cells was abolished (Figure 5D, left). Consistently, the decoy (Figure 5D, right) and knockdown of *OIP5-AS1* (Supplementary Figure S5A) also markedly inhibited p53 binding to the P-site, whereas they did not affect p53 binding to the p21 promoter (Supplementary Figure S5A). Collectively, these results support the hypothesis that *OIP5-AS1* binds to the O-site via its 24-nt region and affects p53 association with the P-site and POX expression when TRPC6 is inhibited.

### Critical role of OIP5-AS1/O-site module in POX transcription and glioma development

**Figure 6:**
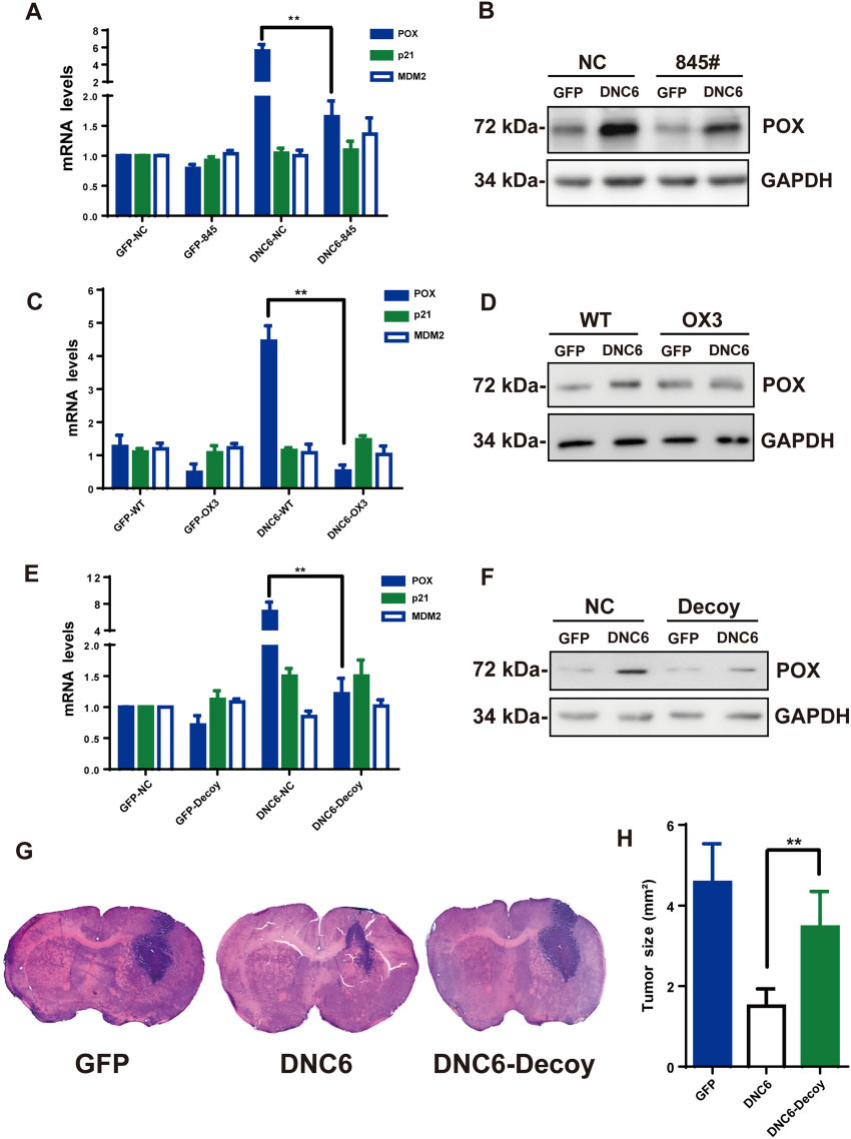
Critical role of *OIP5-AS1*/module in POX transcription and glioma development. (**A** and **B**) POX, p21, and MDM2 mRNA levels in LN229 cells transfected with GFP or DNC6, together with NC (scramble) or 845#. (**C** and **D**) POX, p21, and MDM2 mRNA levels (**C**) and POX protein levels (**D**) in LN229 (WT) or OX3 cells transfected with GFP or DNC6. (**E** and **F**) POX, p21, and MDM2 mRNA levels (**E**) and POX protein levels (**F**) in LN229 cells transfected with GFP or DNC6 with or without the decoy. (**G**) Hematoxylin and eosin staining after 4 weeks of intracranial implantation of glioma cells transfected with GFP, DNC6, or DNC6 with the decoy to nude mice. (**H**) Quantification of the implanted tumor sizes. Unless stated, data are mean ± SEM of at least three independent experiments in triplicate, *t*-test, **P < 0.01.

We next examined the effects of nuclear *OIP5-AS1* knockdown on DNC6-induced POX expression. As shown in Figure 6 and Supplementary Figure S6, DNC6 induced POX upregulation at both the mRNA and protein levels, which was greatly suppressed by 845# (Figure 6A and B; Supplementary Figure S6A) and the decoy (Figure 6E and F; Supplementary Figure S6C), whereas the expression of p21 and MDM2 was not changed. In OX3 cells, POX upregulation was not observed at either the mRNA or protein level (Figure 6C and D; Supplementary Figure S6B). Together, these results suggest that *OIP5-AS1* binding to the O-site is necessary for POX transcription specifically induced by DNC6.

In an established intracranially xenografted glioma nude mouse model, after 28 days of implantation, coronal sections of the brains were stained with hematoxylin and eosin. As shown in Figure 6G and H, tumors from DNC6-infected cells were visibly smaller than those from GFP-infected cells. However, in the presence of the decoy, the tumor size was almost the same as that from the GFP group. These results are consistent with the hypothesis that *OIP5-AS1* binds to the O-site to control POX expression to suppress glioma development.

## Discussion

TFs via their DBDs bind to DNA consensus motifs to control gene expression. Nevertheless, gene expression is temporally and spatially specific. Therefore, it is important to understand what directs a TF to bind to its DNA consensus sequence in one gene promoter but not another. In this study, we report that *OIP5-AS1* determines p53-driven POX transcription as regulated by TRPC6 in glioma. This is supported by several experimental results. First, the inhibition of TRPC6 specifically upregulated POX expression. Second, the p53/OIP5-AS1 complex was pivotal for TRPC6 regulation of POX expression. Third, *OIP5-AS1* via its 24-nt motif binding to the O-site in the POX promoter controlled POX expression. Finally, the *OIP5-AS1*/O-site module was crucial for p53 binding to the POX promoter, POX expression, and glioma growth. Our results thus revealed a new mechanism by which *OIP5-AS1* through complementary sequence pairing can direct p53 to the POX promoter to control its expression. Therefore, lncRNAs and TFs through sequence specificity can regulate gene expression specifically.

The range of ncRNAs in eukaryotes exceeds the number of protein-coding genes (Fatica and Bozzoni, 2014), implying their important roles in cellular activities. Different mechanisms account for a variety of functions of lncRNAs, which can function as competing binding factors (Zhuo et al., 2019) and ‘miRNA sponges’ (Fatica and Bozzoni, 2014). The evidence so far indicates that most nuclear lncRNAs function by guiding chromatin modifiers or TFs to specific genomic loci (Wang et al., 2011; Zhu et al., 2018). Here, we found that a lncRNA, *OIP5-AS1*, can function as a guide to recruit a TF, p53, to a gene promoter by base pairing between the lncRNA and DNA. This may be a new mechanism to explain the precise regulation of gene expression. Indeed, lncRNA binding to gene promoters has been reported (Guo et al., 2018; Zhu et al., 2018). For instance, as a VIM head-to-head antisense transcript on VIM mRNA, VIM-AS1 can form an R-loop with the VIM promoter to regulate NF-κB binding to its promoter and expression (Boque-Sastre et al., 2015). But it is not clear whether VIM-AS1 regulates the expression of other NF-κB-driven genes. Here, we found that *OIP5-AS1* is not an antisense transcript for POX mRNA, but rather helps p53 to bind to the POX promoter via the O-site, thereby specifically regulating POX but not other p53-driven genes. Although the structure of the lncRNA suggests that it has the potential to bind to RNA or DNA by base pairing, the cocrystallization of the lncRNA and DNA could provide further structural information on their binding.

TFs control gene transcription through the interaction between their DBDs and consensus elements in the gene promoter (Maxwell and Kochevar, 2008). In this context, lncRNAs promote gene transcription likely independent of the consensus elements to which TFs bind. Here, the O-site to which *OIP5-AS1* binds is not the p53 consensus element, but it is crucial for p53 binding and p53-driven POX expression. Therefore, in addition to the consensus element, another domain in the promoter is also required for lncRNA-regulated gene expression. In our study, the combination of these two sites, O-site and P-site, determines the specific regulation of POX expression. At present, we do not know how the *OIP5-AS1*/O-site module affects the p53/P-site module. It has been reported that p53 can form a tetramer to control gene expression (Joerger and Fersht, 2008). It is thus speculative that the *OIP5-AS1*/O-site module affects the p53/P-site module via affecting the formation of p53 tetramer.

The intrinsic nucleic acid nature of lncRNAs confers on their dual abilities to bind with RNA/DNA and proteins (Fatica and Bozzoni, 2014). Here, *OIP5-AS1* interacted with both p53 and the O-site in the POX promoter. These findings indicate that *OIP5-AS1* acts as a liaison to hook p53 and the POX promoter together. The question then is whether the part of p53 that *OIP5-AS1* interacts with is the same that interacts with the consensus element. Detailed analysis of the interaction domains between *OIP5-AS1* and p53 will shed light on this question.

An important observation of the current study is that TRPC6, a nonselective cation channel that is permeable to Ca^2+^ (Chen et al., 2019), regulated the interaction between p53 and *OIP5-AS1*. It has been reported that RNA modifications, such as 2ʹ-O-methylation (2ʹ-OMe or N_m_), can affect RNA‒protein interactions (Roundtree et al., 2017). It is therefore speculative that Ca^2+^ influx via TRPC6 can regulate *OIP5-AS1* modifications to control its interaction with p53.

In conclusion, we identified a lncRNA *OIP5-AS1*, which specifically affects POX transcription in a manner that is dependent on its pairing with DNA and its interaction with p53. We revealed that the O-site in the POX promoter is crucial for *OIP5-AS1*-regulated POX expression (Supplementary Figure S6D), but how the O-site exchanges signals with the P-site still remains unclear. In an accompanying work, we provide evidence to demonstrate that the *OIP5-AS1*/O-site and p53/P-site coordinately regulate POX expression in response to TRPC6 signaling in glioma. Specifically, we show that the P-site is crucial for p53-directed H3K9Me2 demethylation at the POX promoter as well as for POX expression specifically regulated by TRPC6 (data not shown).

## Materials and methods

### Key resources of antibodies and cell lines (Table 1)

**Table 1:** Antibodies and cell lines.

Reagent	Resource	Identifier
Antibodies		
POX antibody	Abcam	ab203875, ab93210
TRPC3 antibody	Alomone	ACC-016
p53 antibody	Santa Cruz	sc-126
p21 antibody	Santa Cruz	sc-6246
TRPC6 antibody	Alomone	ACC-017
PTEN antibody	Santa Cruz	sc-7974
GAPDH antibody	Santa Cruz	sc-47724
Cell lines		
LN229	ATCC	LN229 MG
H1299	ATCC	NCI-H1299
U251	ATCC	U251 MG
293T	ATCC	HEK293T

### Cell culture

LN229 (p53^+/–^), U251 (p53-R273H), and H1299 (p53-null) cells maintained in DMEM (Gibco) supplemented with 10% FBS (Four Seasons) and 50 units/ml penicillin and streptomycin (Invitrogen) were cultured in a humidified incubator at 37°C and 5% CO_2_. Transfection was performed using Lipofectamine2000 (Life Technologies) according to the manufacturer’s instructions (Ribobio). *In vivo* morpholino (decoy) was synthesized by Gene tools.

### Adenovirus and lentivirus production and infection

For virus production, 5 × 10^6^ or 1 × 10^7^ HEK293T cells were plated for each 10-cm or 15-cm plate, respectively. The following day, plasmid encoding lentivirus was cotransfected with GFP and DNC6 into the cells using Lipofectamine2000 (Life Technologies) according to the manufacturer’s instructions. The supernatant containing viral particles was collected 48 h after transfection and filtered. For virus infection, cells were infected with virus for 48 h and used for further experiments.

### Human datasets

The data of mRNA expression levels of TRPC6, TP53, PTEN, and POX (PRODH) in human glioma obtained from TCGA database were analyzed by using the cBioPortal for Cancer Genomics (Cerami et al., 2012; Gao et al., 2013).

### Human tissues

Surgically removed human glioma tissues and normal brain tissues were obtained frozen or paraffin-embedded from Changzheng Hospital (Shanghai, China). Gliomas were graded by the Pathology Department of Changzheng Hospital based on the World Health Organization grading system. Human normal brain tissues (mostly from the cortex) were obtained from patients with physical injuries to the brain. These specimens were collected with appropriate informed consent from the patients. The use of human tissue samples was approved by the ethics committee of the hospital.

### Western blotting

Briefly, 20 μg of total proteins collected in 1% SDS RIPA buffer from cell samples were separated by SDS‒PAGE and electrophoretically transferred to a PVDF membrane (Amersham). The membrane was incubated with PBS supplemented with 5% skimmed milk (room temperature, 1 h), primary antibody (4°C, overnight), and then peroxidase-linked secondary antibody (room temperature, 1.5 h). The protein bands were visualized by the SuperSignal West Pico Chemiluminescent substrate toolkit (Pierce) and photographed by chemiluminescent camera (Tiangen). β-actin and GAPDH were the internal controls. Each experiment was performed in triplicate and each measurement was done in triplicate.

### Immunohistological staining

For immunohistological staining, 5-µm thick paraffin-processed sections were mounted on poly-D-lysine–coated glass slides. Each slide was dewaxed in 100% xylene and rehydrated by incubation in decreasing concentrations (100%, 95%, and 75%) of alcohol. Sections were incubated in 3% hydrogen peroxide to quench endogenous peroxidase for >20 min. Sections were digested in antigen retrieval buffer (50 mM, Tris–HCl, pH 8.0, 5 mM EDTA) with proteinase K (Merck KGaA) at a final concentration of 10 µg/ml. The immunoreactions were performed with VECTASTAIN Elite ABC Kit (Vector Laboratories). Briefly, sections were blocked with horse serum and incubated overnight with diluted antibodies against POX or GFAP at 4°C. Following washes with PBS, the slides were incubated with the appropriate biotinylated anti-rabbit IgG secondary antibodies (Vector Laboratories) at room temperature for 2 h, washed, and incubated with horseradish peroxidase-conjugated streptavidin (Vector Laboratories). Sections were developed using a peroxidase substrate 3,3′-diaminobenzidine kit (Vector Laboratories) and counterstained with hematoxylin to stain the nucleus. Sections were dehydrated by incubation in increasing concentrations (75%, 95%, and 100%) of alcohol and in 100% xylene before coverslips were mounted onto the sections. POX immunostaining was measured based on the relative optical intensity of specific area of the brown-colored 3,3′-diaminobenzidine signal.

### Reverse transcription and qPCR

Total RNA extracted from human tissues, U87, LN229, and U251 cells using Trizol reagent (Life Technologies) was reverse-transcribed to the first-strand cDNA in a 20-µl reaction volume containing 5 µg RNA, random primer, dNTP, RNAase inhibitor, and M-MuLV reverse transcriptase (Promega) for 25°C, 10 min, then 42°C, 1.5 h, and 72°C, 10 min. qPCR was performed with SYBR Premix ExTaq Kit (Genestar) and analyzed on Step One plus (ABI). The qPCR conditions were as follows: 95°C, 10 min; 95°C, 10 sec, 60°C, 15 sec, 72°C, 20 sec, and additional 39 cycles. Quantification was done by using the comparative Ct (ΔΔCt) method. The primers used for qPCR are listed in Supplementary Table S1.

### In situ hybridization

The three probes targeting the human POX mRNA (GenBank accession NM_016335.4) were designed and made by Boster Company. The sequences were: (i) ACGAATAAGCGGGACAAGCAATACCAGGCCCACCG; (ii) AAGTGGAGGTGCTTCTTTCACCAAATGGCTGTGGA; (iii) TTCAACACATACCAGTGCTACCTCAAGGATGCCTA.

Both the sense and antisense probes were labelled with digoxigenin. Hybridization was performed on 8-µm cryosections of freshly frozen tissues. Sections were immersed in the hybridization solution, containing 50% formamide, 5× saline sodium citrate (SSC) buffer (0.75 M NaCl and 75 mM sodium citrate, pH 7.0), 50 µg/ml yeast tRNA, 100 µg/ml heparin, 1× Denhardt solution, 0.1% 3-[(3-cholamidopropyl) dimethylammonio]-1-propanesulfonate, 0.1% Tween-20, and 5 mM EDTA, and hybridized at 65°C for 16 h with sense or antisense probe at a concentration of 1 µg/ml. Post-hybridization washes were performed three times in 2× SSC at 37°C for 5 min and twice in 0.2× SSC at 65°C for 30 min. The hybridized probes were detected using alkaline phosphatase-conjugated anti-digoxigenin Fab fragments and sections were developed using a peroxidase substrate 3,3′-diaminobenzidine kit.

### ChIP

The ChIP assays were performed according to the previous report (Zhang et al., 2016). Briefly, 1 × 10^7^ cells were washed with PBS, fixed with 1% formaldehyde, and quenched with 0.125 M glycine. Cells were then collected for nuclear extraction by 400 μl lysis buffer (50 mM, Tris‒HCl, pH 7.4, 1% SDS, 10 mM EDTA) and 120 µg of sonicated chromatin (10 sec sonication, 4 times until the lysates turned clear and then supernatant was collected) were incubated with 10 µg antibodies against p53, POL II, or IgG (Santa Cruz) at 4°C, overnight. The next day, the protein A/G magnetic beads (Biotool, b23202; preblocked with 10 mg/ml BSA and 70 µg/ml salmon sperm DNA, overnight) were added, incubated for 2 h at 4°C, and then collected by magnetic scaffold. The precipitated beads were sequentially washed three times with the SB140 buffer (50 mM HEPES, pH 7.9, 1 mM EDTA, 1% Triton X-100, 0.1% SDS, 140 mM NaCl, 0.1% deoxycholate), the SB500 buffer (50 mM HEPES, pH 7.9, 1 mM EDTA, 1% Triton X-100, 0.1% SDS, 500 mM NaCl, 0.1% deoxycholate), the LiCl buffer (20 mM, Tris‒HCl, pH 8.0, 1 mM EDTA, 250 mM LiCl, 0.5% deoxycholate, 0.5% Nonidet P-40), and the TE buffer (20 mM, Tris‒HCl, pH 8.0, 1 mM EDTA) for 4 min each with rotation. Then, the beads were eluted by the elution buffer (50 mM, Tris‒HCl, pH 8.0, 1% SDS, 10 mM EDTA) and TE‒SDS buffer (1% SDS) and incubated at 65°C, overnight. The eluted beads were then digested by PK buffer (0.4 μg/μl protease K in TE buffer) for at least 4 h. Relative enrichment of the interest targets in the precipitates was measured by qPCR. The p53 and POL II signals were normalized to input and IgG. The fold change relative to the control cells was then calculated. Rabbit and mouse IgG (Santa Cruz) were used as nonspecific IgG controls.

### RIP

All solutions were in DEPC-treated H_2_O. Briefly, cells were washed with PBS and fixed by 1% formaldehyde in cold PBS for 10 min and quenched with 0.125 M glycine. Cells were collected in EP tubes and washed with PBS twice, suspended by lysis buffer (50 mM, Tris‒HCl, pH 7.4, 1% SDS, 10 mM EDTA). After a freezing and thawing cycle in nitrogen, the cell lysates were treated by 30 min ultrasonication until they turned clear. The antibodies against IgG or p53 were added into the collected supernatant and incubated overnight at 4°C. The protein A/G magnetic beads (preblocked with 10 mg/ml BSA and 70 μg/ml yeast tRNA, overnight) were added and incubated for 2 h at room temperature and centrifuged. The precipitates were sequentially washed by wash buffer I (50 mM, Tris‒HCl, pH 7.5, 1 M NaCl, 1% Nonidet P-40, 1% deoxycholate, 1 M urea) for 5 min, 3 times and wash buffer II (50 mM, Tris‒HCl, pH 7.5, 1 M NaCl, 1% Nonidet P-40, 1% deoxycholate) for 5 min, 3 times and sequentially eluted by elution buffer and same volume of TES buffer (50 mM, Tris‒HCl, pH 7.4, 10 mM EDTA) at 55°C for 15 min. Then, total RNA in eluted products was extracted by TRIZOL and transcribed to cDNA for qPCR analysis.

### Nuclear extraction

LN229 cells were washed with ice-cold PBS and resuspended in ice-cold buffer A containing 10 mM HEPES, pH 7.9, 10 mM KCl, 0.1 mM EDTA, 1 mM dithiothreitol (DTT), 1% sodium deoxycholate, and protease inhibitor cocktail. The cells were incubated on ice for 10 min and 0.5% Nonidet P-40 (Sangon) for additional 15 min. After centrifuging at 6000 *g* for 1 min, the pellet was resuspended in extract buffer (20 mM HEPES, pH 7.9, 400 mM KCl, 4.5 mM MgCl_2_, 0.2 mM EDTA, 1 mM DTT, 1% sodium deoxycholate, and protease inhibitor cocktail) and incubated on ice for 1 h. The lysate was centrifuged at 10000 *g* for 10 min, and the sediment was collected as nuclear extracts.

### EMSA

Briefly, synthetic DNA oligos and *in vitro* transcription of lncRNA were mixed in hybridization buffer (50 mM, Tris‒HCl, pH 8.0, 250 mM NaCl, 2.5 mM EDTA, 2.5 mM DTT, 5 mM MgCl_2_) for 2 h, from 95°C to 25°C, gradually. The formation of DNA/RNA hybrid was visualized in 1% agarose gel stained with nucleic acid loading dyes. Human p53 protein was purified (Detai Bio) and mixed with DNA or RNA in hybridization buffer at 37°C for 1 h. Then, the formation of DNA/protein hybrid or RNA/protein hybrid was visualized by 1% agarose gel stained with nucleic acid loading dyes.

### ChIRP

The ChIRP was conducted following the protocol as previously reported (Chu et al., 2011). Briefly, Cells collected were fixed by cold 1% glutaraldehyde for 10 min and quenched with 0.125 M glycine for 5 min. They were immediately put into liquid nitrogen and then 400 μl lysis buffer (50 mM, Tris‒HCl, pH 7.4, 1% SDS, 10 mM EDTA) per 10^7^ cells were added into the cells for ∼30 min ultrasonication until lysates turned clear. Then, 20 μl lysates were used as total DNA or RNA input, respectively. After addition of 2 pmol *OIP5-AS1* probes, hybridization was made at 37°C for 4 h with shaking and 20 µl Dyn-2 magnetic beads (Life Technologies) per sample were added and incubated at 37°C for 30 min with shaking. Then, the beads were washed 5 times with wash buffer (2× SSC, 0.5% SDS, phenylmethanesulfonyl fluoride) and eluted by 150 µl elution buffer (50 mM, Tris‒HCl, pH 8.0, 1% SDS, 10 mM EDTA) at 37°C for 30 min with shaking. The lysates were transferred to a fresh 1.5-ml RNAase-free EP tube and 90 μl PK buffer (100 mM NaCl, 10 mM, Tris‒HCl, pH 8.0 for DNA or pH 7.0 for RNA, 1 mM EDTA, 0.5% SDS) and 5% protease K (20 mg/ml) were added and incubated at 50°C for 45 min. The eluted products were then used for DNA, RNA, or protein analysis.

### POX promoter editing by CRISPR/Cas9

CRISPR/Cas9 target site was searched within the O-site in POX promoter region 3000 nt upstream of transcription start site. The Cas9 guide sequence was designed by online CRISPR Design Tool (http://tools.genome-engineering.org) (Ran et al., 2013). Guide sequences were selected for cell genome editing (Target sg-RNA 1: 5′-TCCCAAGTAGCTAGGATTAC-3′; Target sg-RNA 2: 5′-TGTAATCCTAGCTACTTGGG-3′), and a 149-bp repair replacement template (single-stranded DNA oligonucleotides) containing mutations in O-site region was designed to target and replace the POX promoter region: 5′-TGATGGCTTAGCTTGGGCTCAGAGGCCTGACACCTGGGCTCAAGTGATCCCGACCGTCCTCGAGCATCTCGCCTCGGGACTACAGGCTGGACTGCACCTAGCTAATTTTTAAAAAATATTTTTTTG-3′. The construction of sg-RNA-expressing plasmids and clone isolation of cell lines were done following the reported protocol (Ran et al., 2013). The plasmid for construction of sg-RNA expression was pSpCas9(BB)-2A-Puro (Addgene plasmid 62988).

### In vivo studies

In the intracranial glioma model, U87 cells infected with adenovirus at MOI of 10 for 24 h were incubated with the decoy (0.1 mM) for 24 h. Then, 5 × 10^5^ cells suspended in 5 µl Leibovitz L-15 medium (without serum or antibiotics and with 2 mM L-glutamine) (Invitrogen) were injected into the right caudate putamen of athymic nude male mice (5-week-old, *n *=* *12 per group). Briefly, a 0.5‒0.8-mm burr hole was made 2 mm right to the midline and 1 mm anterior to the bregma before the cells were stereotactically injected by a 50-µl syringe and 23-gauge needle (0.6-mm diameter) to a depth of 3.9 mm. Cells were injected at a speed of 1 µl/min. After 4 weeks of implantation, mice were anesthetized with chloral hydrate and perfused with 4% paraformaldehyde, and their brains were removed for cryosections at 25 µm per slice for hematoxylin and eosin staining. The implanted tumor size was quantified by calculating average areas of four continuous serial coronal cryosections of each brain. All athymic nude mice were kept under specific pathogen-free conditions and their care was in accord with the animal welfare guidelines.

### Quantification and statistical analysis

Statistics was carried out using Microsoft Excel and GraphPad Prism to evaluate the differences between different groups, using the *t*-test or ANOVA (for more than two groups). All results are shown as mean ± SEM from three or more independent experiments. *P*-values <0.05 were considered statistically significant, *P < 0.05, **P < 0.01, ***P < 0.001, ****P < 0.0001, respectively.

## Supplementary material

Supplementary material is available at *Journal of Molecular Cell Biology* online.

## Supplementary Material

mjab001_Supplementary_Data

## References

[mjab001-B1] Beckerman R., Prives C. (2010). Transcriptional regulation by p53. Cold Spring Harb. Perspect. Biol. 2, a000935. 20679336 10.1101/cshperspect.a000935 PMC2908772

[mjab001-B2] Boque-Sastre R., Soler M., Oliveira-Mateos C., et al (2015). Head-to-head antisense transcription and R-loop formation promotes transcriptional activation. Proc. Natl Acad. Sci. USA 112, 5785–5790. 25902512 10.1073/pnas.1421197112 PMC4426458

[mjab001-B3] Cai R., Ding X., Zhou K., et al (2009). Blockade of TRPC6 channels induced G2/M phase arrest and suppressed growth in human gastric cancer cells. Int. J. Cancer 125, 2281–2287. 19610066 10.1002/ijc.24551

[mjab001-B4] Cerami E., Gao J., Dogrusoz U., et al (2012). The cBio cancer genomics portal: an open platform for exploring multidimensional cancer genomics data. Cancer Discov. 2, 401–404. 22588877 10.1158/2159-8290.CD-12-0095 PMC3956037

[mjab001-B5] Chen J., McKay R.M., Parada L.F. (2012). Malignant glioma: lessons from genomics, mouse models, and stem cells. Cell 149, 36–47. 22464322 10.1016/j.cell.2012.03.009 PMC3719882

[mjab001-B6] Chen J.M., Li Q.W., Liu J.S., et al (2019). TRPC6 mRNA levels in peripheral leucocytes of patients with Alzheimer's disease and mild cognitive impairment: a case-control study. Prog. Neuropsychopharmacol. Biol. Psychiatry 92, 279–284. 30684527 10.1016/j.pnpbp.2019.01.009

[mjab001-B7] Chigurupati S., Venkataraman R., Barrera D., et al (2010). Receptor channel TRPC6 is a key mediator of Notch-driven glioblastoma growth and invasiveness. Cancer Res. 70, 418–427. 20028870 10.1158/0008-5472.CAN-09-2654

[mjab001-B8] Chiu H.S., Somvanshi S., Patel E., et al (2018). Pan-cancer analysis of lncRNA regulation supports their targeting of cancer genes in each tumor context. Cell Rep. 23, 297–312.e12. 29617668 10.1016/j.celrep.2018.03.064 PMC5906131

[mjab001-B9] Chu C., Qu K., Zhong F.L., et al (2011). Genomic maps of long noncoding RNA occupancy reveal principles of RNA-chromatin interactions. Mol. Cell 44, 667–678. 21963238 10.1016/j.molcel.2011.08.027 PMC3249421

[mjab001-B10] Ding X., He Z., Zhou K., et al (2010). Essential role of TRPC6 channels in G2/M phase transition and development of human glioma. J. Natl Cancer Inst. 102, 1052–1068. 20554944 10.1093/jnci/djq217

[mjab001-B11] El Boustany C., Bidaux G., Enfissi A., et al (2008). Capacitative calcium entry and transient receptor potential canonical 6 expression control human hepatoma cell proliferation. Hepatology 47, 2068–2077. 18506892 10.1002/hep.22263

[mjab001-B12] Fatica A., Bozzoni I. (2014). Long non-coding RNAs: new players in cell differentiation and development. Nat. Rev. Genet. 15, 7–21. 24296535 10.1038/nrg3606

[mjab001-B13] Gao J.J., Aksoy B.A., Dogrusoz U., et al (2013). Integrative analysis of complex cancer genomics and clinical profiles using the cBioPortal. Sci. Signal. 6, pl1. 23550210 10.1126/scisignal.2004088 PMC4160307

[mjab001-B14] Giaccone G., Battey J., Gazdar A.F., et al (1992). Neuromedin B is present in lung cancer cell lines. Cancer Res. 52 *(9 Suppl)*, 2732s–2736s. 1563005

[mjab001-B15] Guo X., Xu Y., Wang Z., et al (2018). A Linc1405/Eomes complex promotes cardiac mesoderm specification and cardiogenesis. Cell Stem Cell 22, 893–908.e96. 29754779 10.1016/j.stem.2018.04.013

[mjab001-B16] Hofmann T., Schaefer M., Schultz G., et al (2002). Subunit composition of mammalian transient receptor potential channels in living cells. Proc. Natl Acad. Sci. USA 99, 7461–7466. 12032305 10.1073/pnas.102596199 PMC124253

[mjab001-B17] Joerger A.C., Fersht A.R. (2008). Structural biology of the tumor suppressor p53. Annu. Rev. Biochem. 77, 557–582. 18410249 10.1146/annurev.biochem.77.060806.091238

[mjab001-B18] Kim J., Abdelmohsen K., Yang X., et al (2016). LncRNA OIP5-AS1/cyrano sponges RNA-binding protein HuR. Nucleic Acids Res. 44, 2378–2392. 26819413 10.1093/nar/gkw017 PMC4797289

[mjab001-B19] Kleaveland B., Shi C.Y., Stefano J., et al (2018). A network of noncoding regulatory RNAs acts in the mammalian brain. Cell 174, 350–362.e17. 29887379 10.1016/j.cell.2018.05.022 PMC6559361

[mjab001-B20] Kopp F., Mendell J.T. (2018). Functional classification and experimental dissection of long noncoding RNAs. Cell 172, 393–407. 29373828 10.1016/j.cell.2018.01.011 PMC5978744

[mjab001-B21] Lambert S.A., Jolma A., Campitelli L.F., et al (2018). The human transcription factors. Cell 172, 650–665. 29425488 10.1016/j.cell.2018.01.029 PMC12908702

[mjab001-B22] Lennox K.A., Behlke M.A. (2016). Cellular localization of long non-coding RNAs affects silencing by RNAi more than by antisense oligonucleotides. Nucleic Acids Res. 44, 863–877. 26578588 10.1093/nar/gkv1206 PMC4737147

[mjab001-B23] Liu Y., Borchert G.L., Donald S.P., et al (2005). MnSOD inhibits proline oxidase-induced apoptosis in colorectal cancer cells. Carcinogenesis 26, 1335–1342. 15817612 10.1093/carcin/bgi083

[mjab001-B24] Ma B., Pan Y., Zheng J., et al (2007). Sequence analysis of p53 response-elements suggests multiple binding modes of the p53 tetramer to DNA targets. Nucleic Acids Res. 35, 2986–3001. 17439973 10.1093/nar/gkm192 PMC1888811

[mjab001-B25] Maxwell S.A., Kochevar G.J. (2008). Identification of a p53-response element in the promoter of the proline oxidase gene. Biochem. Biophys. Res. Commun. 369, 308–313. 18279664 10.1016/j.bbrc.2008.01.171

[mjab001-B26] May P., May E. (1999). Twenty years of p53 research: structural and functional aspects of the p53 protein. Oncogene 18, 7621–7636. 10618702 10.1038/sj.onc.1203285

[mjab001-B27] Nishida M., Kuwahara K., Kozai D., et al (2015). TRP channels: their function and potentiality as drug targets. In: Nakao, K., Minato, N., and Uemoto, S. (eds). Innovative Medicine: Basic Research and Development. Tokyo: Springer, 195–218. 29787169

[mjab001-B28] Phang J.M. (2019). Proline metabolism in cell regulation and cancer biology: recent advances and hypotheses. Antioxid. Redox Signal. 30, 635–649. 28990419 10.1089/ars.2017.7350 PMC6338564

[mjab001-B29] Polyak K., Xia Y., Zweier J.L., et al (1997). A model for p53-induced apoptosis. Nature 389, 300–305. 9305847 10.1038/38525

[mjab001-B30] Ponting C.P., Oliver P.L., Reik W. (2009). Evolution and functions of long noncoding RNAs. Cell 136, 629–641. 19239885 10.1016/j.cell.2009.02.006

[mjab001-B31] Ran F.A., Hsu P.D., Wright J., et al (2013). Genome engineering using the CRISPR–Cas9 system. Nat. Protoc. 8, 2281–2308. 24157548 10.1038/nprot.2013.143 PMC3969860

[mjab001-B32] Riley T., Sontag E., Chen P., et al (2008). Transcriptional control of human p53-regulated genes. Nat. Rev. Mol. Cell Biol. 9, 402–412. 18431400 10.1038/nrm2395

[mjab001-B33] Roundtree I.A., Evans M.E., Pan T., et al (2017). Dynamic RNA modifications in gene expression regulation. Cell 169, 1187–1200. 28622506 10.1016/j.cell.2017.05.045 PMC5657247

[mjab001-B34] Shaw P.H. (1996). The role of p53 in cell cycle regulation. Pathol. Res. Pract. 192, 669–675. 8880867 10.1016/S0344-0338(96)80088-4

[mjab001-B35] Summerton J. (1999). Morpholino antisense oligomers: the case for an RNase H-independent structural type. Biochim. Biophys. Acta 1489, 141–158. 10807004 10.1016/s0167-4781(99)00150-5

[mjab001-B36] Ulitsky I., Shkumatava A., Jan C.H., et al (2011). Conserved function of lincRNAs in vertebrate embryonic development despite rapid sequence evolution. Cell 147, 1537–1550. 22196729 10.1016/j.cell.2011.11.055 PMC3376356

[mjab001-B37] Wang K.C., Yang Y.W., Liu B., et al (2011). A long noncoding RNA maintains active chromatin to coordinate homeotic gene expression. Nature 472, 120–124. 21423168 10.1038/nature09819 PMC3670758

[mjab001-B38] Zhang Y., Zhang D., Li Q., et al (2016). Nucleation of DNA repair factors by FOXA1 links DNA demethylation to transcriptional pioneering. Nat. Genet. 48, 1003–1013. 27500525 10.1038/ng.3635

[mjab001-B39] Zhu P., Wu J., Wang Y., et al (2018). LncGata6 maintains stemness of intestinal stem cells and promotes intestinal tumorigenesis. Nat. Cell Biol. 20, 1134–1144. 30224759 10.1038/s41556-018-0194-0

[mjab001-B40] Zhuo W., Liu Y., Li S., et al (2019). Long noncoding RNA GMAN, up-regulated in gastric cancer tissues, is associated with metastasis in patients and promotes translation of ephrin A1 by competitively binding GMAN-AS. Gastroenterology 156, 676–691.e11. 30445010 10.1053/j.gastro.2018.10.054

