# Supplementary material for: OIP5-AS1 specifies p53-driven POX transcription regulated by TRPC6 in glioma
Source: J Mol Cell Biol. 2021 Jan 28;13(6):409–21. doi: 10.1093/jmcb/mjab001 (PMC8436707; doi:10.1093/jmcb/mjab001)
Supplement: mjab001_Supplementary_Data [file mjab001_supplementary_data.pdf]

Supplementary material

OIP5-AS1 specifies p53-driven POX transcription regulated by TRPC6 in glioma

Wei Shao[1,†], Zhen-Yu Hao[1,†], Yi-Fei Chen[1,†], Jun Du[1], Qian He[1], Liang-Liang Ren[1], Yan Gao[1], Nan Song[1], Yan Song[1], Hua He[2], and Yi-Zheng Wang[1,*]

[1] The Brain Science Center, Beijing Institute of Basic Medical Sciences, Beijing 100850, China

[2] Department of Neurosurgery, Changzheng Hospital, Second Military Medical University, Shanghai 200003, China

[†] These authors contributed equally to this work.

* Correspondence to: Yi-Zheng Wang, E-mail: yzwang@ion.ac.cn

Supplemental figure 1

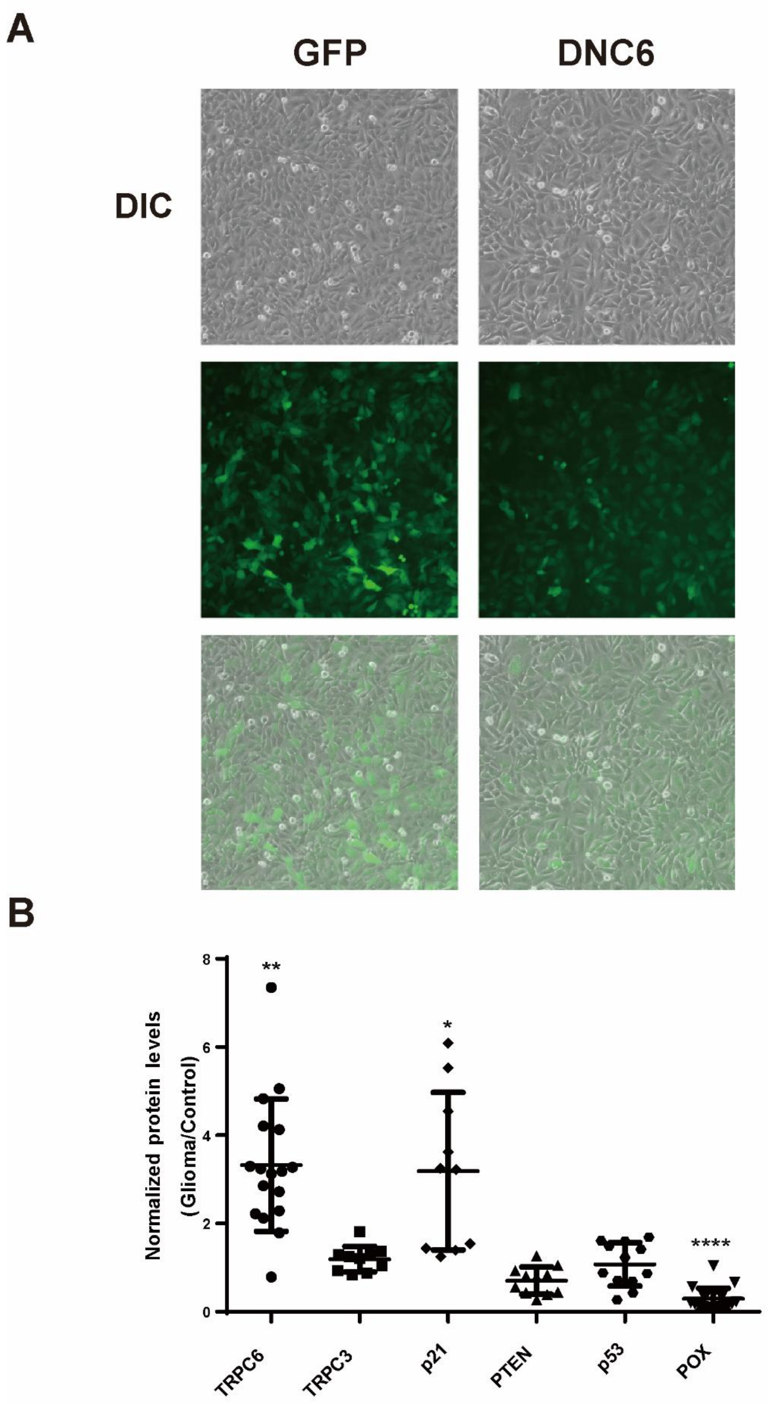

Supplementary Figure S1

(A) DIC and fluorescent image of LN229 cells transfected with adenovirus GFP or DNC6-GFP. **(B)** Quantification of protein levels of TRPC6, TRPC3, p21, p53, PTEN and POX in glioma and normal brain tissues (*$P < 0.05$, **$P < 0.01$, ****$P < 0.0001$, *t-test*). Unless stated, data are means ± SD of at least three independent experiments in triplicate.

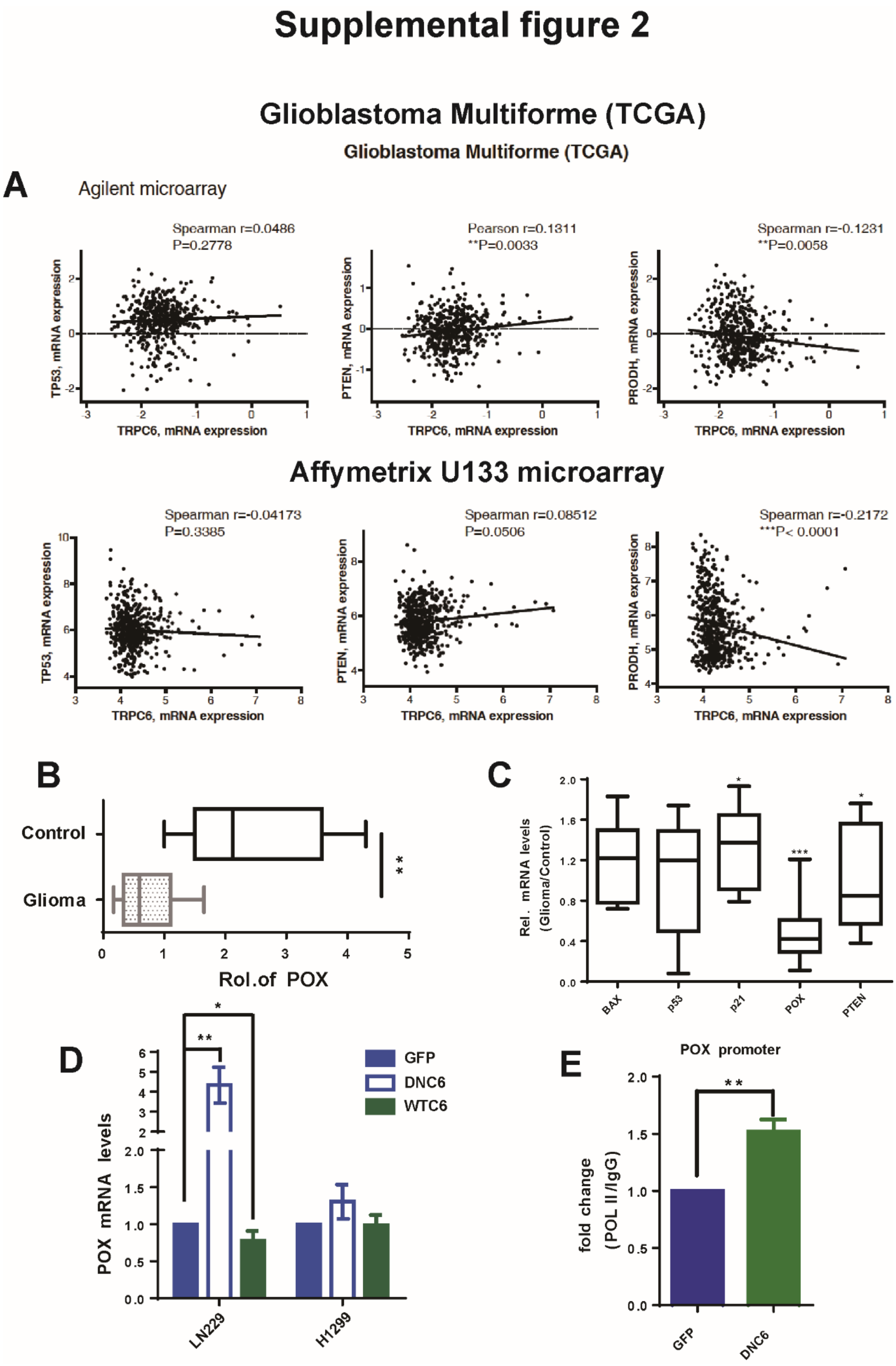

Supplementary Figure S2

(A) Correlation analysis of TRPC6 and PTEN, p53 and POX(PRODH) using TCGA data. Upper panel: Agilent microarray data; lower panel: Affymetrix U133 microarry data. **(B)** Quantification of POX immunostaining of DAB optic bright signal in glioma and normal (control) brain tissues. ROI; region of interest (**P < 0.01, *t-test*). **(C)** qPCR analysis of BAX, p53, p21, POX and PTEN mRNA levels in glioma or normal brain tissues (*P < 0.05, ***P < 0.001, *t-test*). **(D)** qPCR analysis of POX

mRNA levels in LN229 cells and H1299 cells transfected with GFP, DNC6 or WTC6 (*P < 0.05, **P < 0.01, *t-test*). **(E)** qPCR analysis of polymerase II (POL-II) levels at POX promoter in POL-II ChIP assay of LN229 cells transfected with GFP or DNC6 (**P < 0.01, *t-test*). Unless stated, data are means ± SEM of at least three independent experiments in triplicate.

Supplemental figure 3

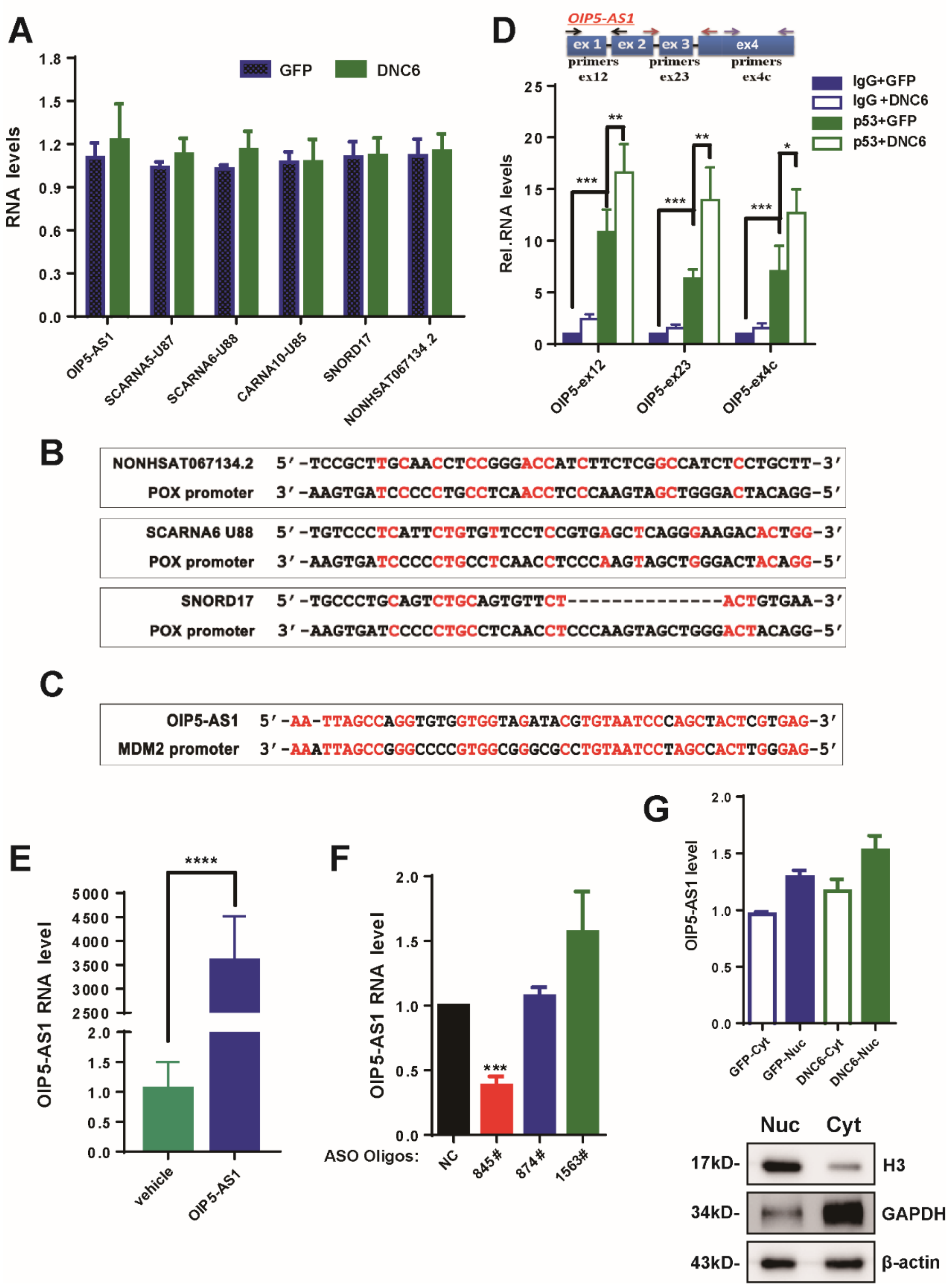

Supplementary Figure S3

(A) qPCR analysis of OIP5-AS1 and other 5 non-coding RNA levels in LN229 cells transfected with GFP or DNC6 after 48hr. **(B)** BLAST analysis of p53-RIP sequencing screened top scored non-coding RNAs and POX promoter (region of 3000nt upstream of transcription start site). **(C)** BLAST analysis of OIP5-AS1 and MDM2 promoter (region of 3000nt upstream of transcription start site). **(D)** qPCR analysis of OIP5-AS1 in p53- or IgG-precipitates from LN229 cells transfected with

GFP or DNC6 (****P* < 0.001, ***P* < 0.01, **P* < 0.05, *t-test*). The ex12, ex23, ex4c represents qPCR primers illustrated in the graphic model. **(E)** qPCR analysis of OIP5-AS1 levels in LN229 cells transfected with OIP-AS1 for 48 hours (*****P* < 0.0001, *t-test*). **(F)** qPCR analysis of OIP5-AS1 levels in LN229 cells transfected with 845#, 874# or 1563# ASO against OIP5-AS1 for 48 hours (****P* <0.001, *t-test*). **(G)** Upper: qPCR analysis of nuclear (Nuc) or cytosolic (Cyt) OIP5-AS1 levels in LN229 cells. Lower: immunoblots of nuclear or cytosolic fractions of LN229 cells using the antibodies against H3, GAPDH or β-actin. Four hours post transfection infected cell by adenovirus. Unless stated, data are means ± SEM of at least three independent experiments in triplicate.

Supplemental figure 4

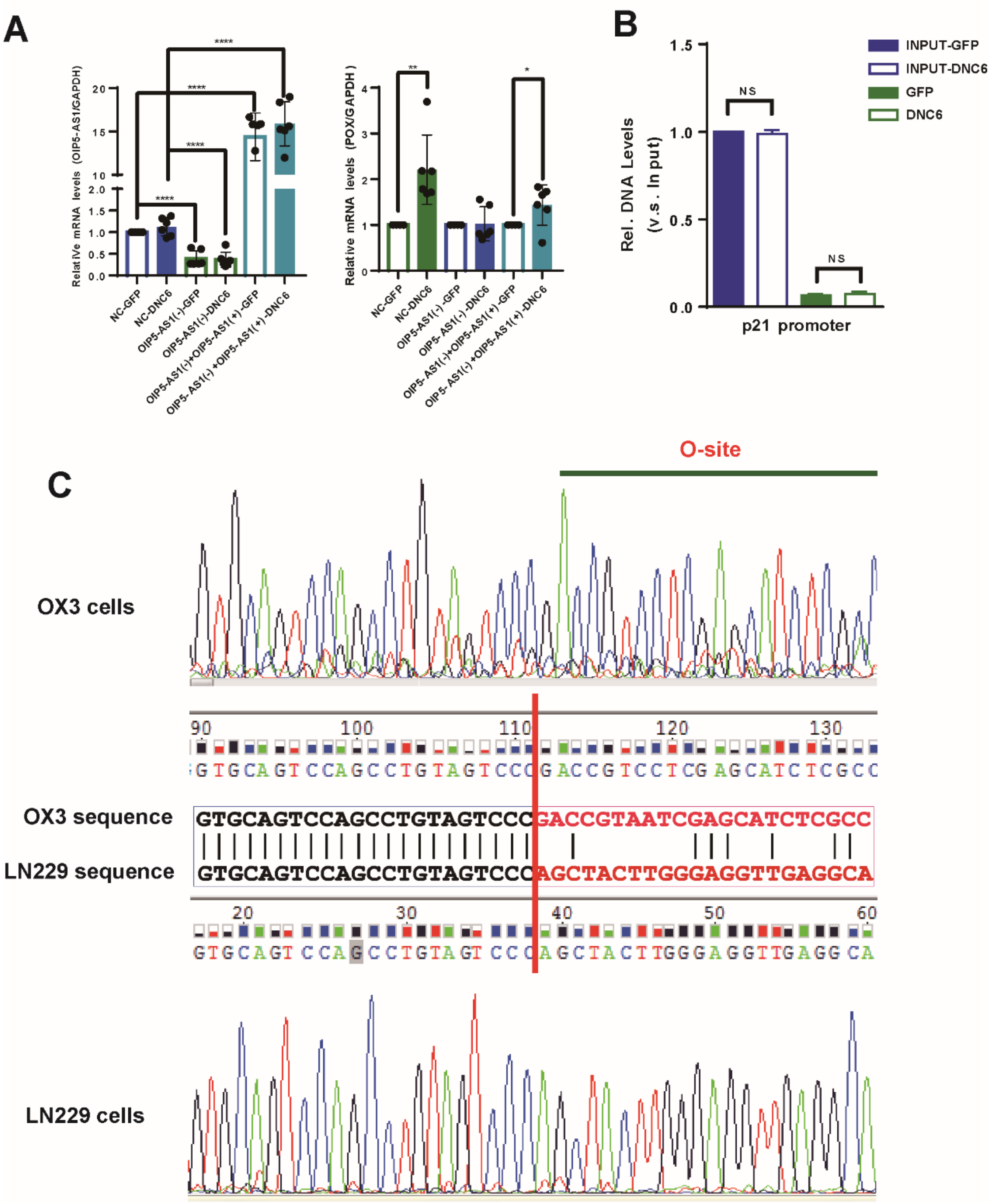

Supplementary Figure S4

(A) qPCR analysis of OIP5-AS1 levels after knockdown of OIP5-AS1 (OIP5-AS1(-)) in LN229 cells. Overexpression of OIP5-AS1 (OIP5-AS1(+)) restored OIP5-AS1 levels in the LN229 cells (Left). qPCR analysis of POX mRNA levels in LN229 cells. Overexpression of OIP5-AS1 (OIP5- AS1(+)) restored DNC6 effects on POX expression in the LN229 cells with knockdown of OIP5- AS1 (OIP5-AS1(-)) (Right) (*****P* < 0.0001, ***P* < 0.01, **P* < 0.05, *t-test*). **(B)** qPCR analysis of p21

promoter DNA levels in the complexes precipitated by 3'-biotin-labeled OIP5-AS1 probe in ChIRP assay. **(C)** Sequencing analysis of the mutation at the O-site in OX3 cell s generated by CRISPR-Cas9. Unless stated, data are means ± SEM of at least three independent experiments in triplicate.

Supplemental figure 5

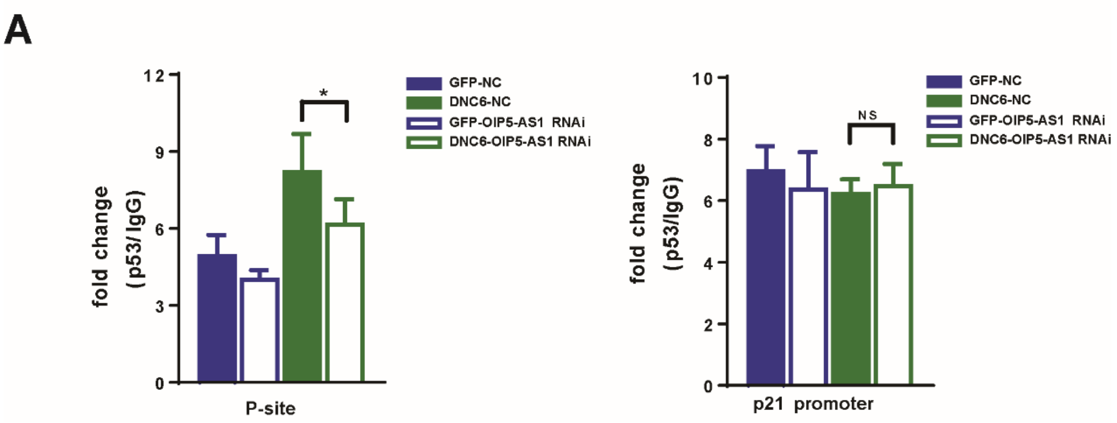

Supplementary Figure S5

(A) qPCR analysis of p53 levels at POX and p21 promoter in ChIP assay in LN229 cells transfected with OIP5-AS1 RNAi (Kim et al., 2016) (**P* < 0.05, *t-test*). Four hours post transfection infected cell by adenovirus. Unless stated, data are means ± SEM of at least three independent experiments in triplicate, NS: no significance.

Reference:

Kim, J., Abdelmohsen, K., Yang, X., et al. (2016). LncRNA OIP5-AS1/cyrano sponges RNA-binding protein HuR. Nucleic Acids Res *44*, 2378-2392.

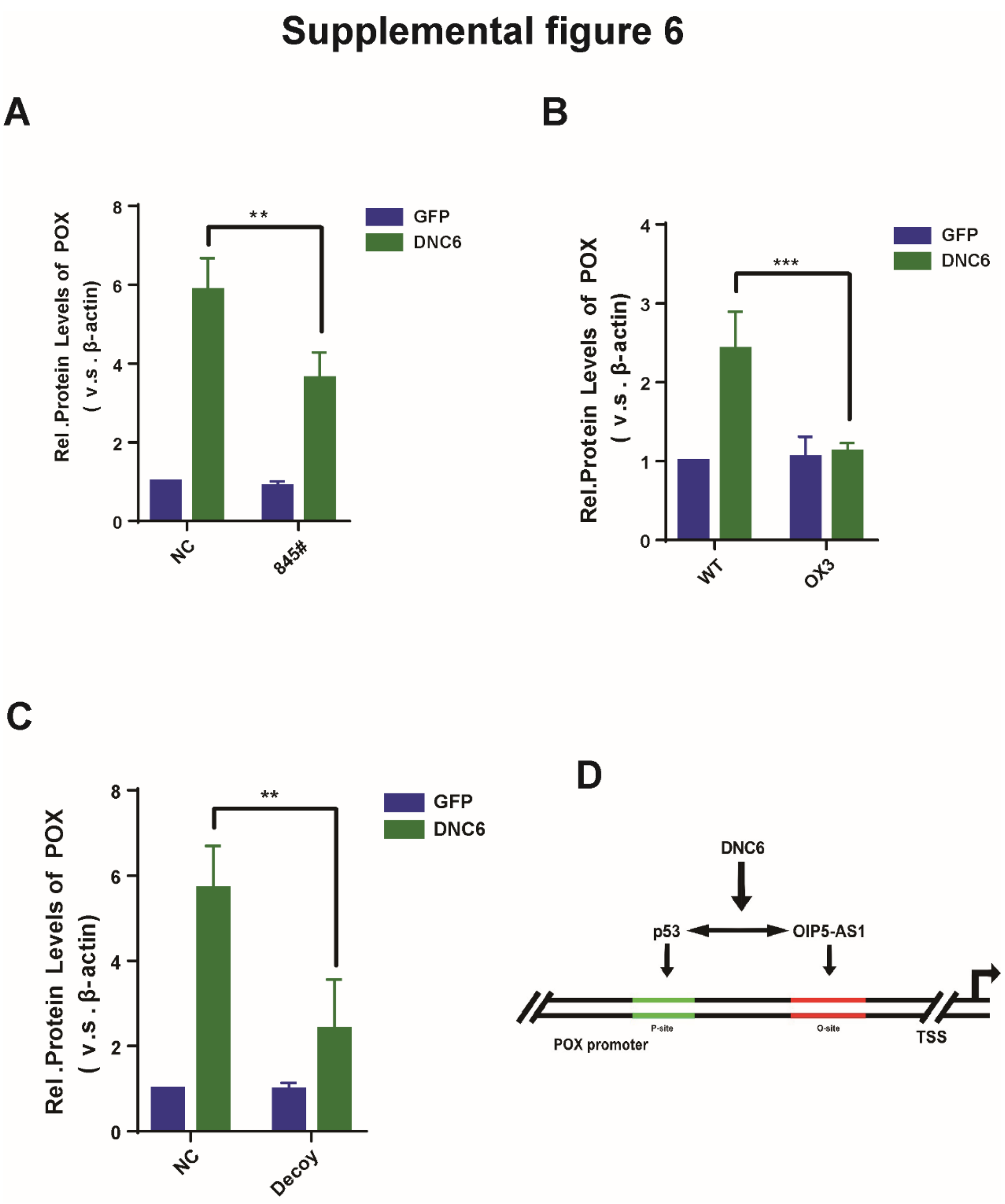

Supplementary Figure S6

(A) Quantification of protein levels of POX in LN229 cells transfected with GFP or DNC6 with NC or antisense oligo 845#, NC: scramble ASO (***P* < 0.01, *t-test*). **(B)** Quantification of protein levels of POX in LN229 (WT) and OX3 cells transfected with GFP or DNC6 (****P* < 0.001, *t-test*). **(C)** Quantification of protein levels of POX in LN229 cells transfected with GFP or DNC6 plus NC or decoy, NC: scramble decoy (***P* < 0.01, *t-test*). Four hours post transfection infected cell by adenovirus. **(D)** A Working model to depict the specific effects of DNC6 on POX expression via p53/OIP5-AS1 complex. Unless stated, data are means ± SEM of at least three independent experiments in triplicate.

Supplementary Table S1 Oligos and primers.

**Oligonucleotide sequences of shRNAs**	
shTRPC6	Forward: 5'-TGGACTTGACGAAAGTAACATTCAAGAGATGT TACTTTCGTCAAGTCCTTTTTTC-3'
	Reverse: 5'-TCGAGAAAAAAGGACTTGACGAAAGTAACATC TCTTGAATGTTACTTTCGTCAAGTCCA-3'
Nonsense shRNA	Forward: 5'-TGTTCTCCGAACGTGTCACGTTCAAGAGACGT GACACGTTCGGAGAACTTTTTTC-3'
	Reverse: 5'-TCGAGAAAAAAGTTCTCCGAACGTGTCACGTC TCTTGAACGTGACACGTTCGGAGAACA-3'
2′OMe-PS ASOs NC	mG*mC*mG*mU*mA*T*T*A*T*A*G*C*C*G*A*mU*mU*mA*mA*mC
2′OMe-PS ASOs 845	mA*mU*mG*mU*mC*A*C*A*G*G*A*T*G*A*G*mC*mC*mA*mG*mG
2′OMe-PS ASOs 1563	mA*mG*mG*mC*mT*G*A*G*C*G*T*G*G*T*G*mG*mT*mG*mC*mG
2′OMe-PS ASOs 874	mT*mT*mG*mG*mG*C*C*T*T*T*G*T*T*C*C*mT*mU*mT*mT*mC
m = 2 OMe; '*' = PS linkage.	
**Primers used in qPCR assays**	
p53	Forward: 5'-GCTTTCCACGACGGTGAC-3'
	Reverse: 5'- GCTCGACGCTAGGATCTGAC-3'
POX	Forward:5'-CCGCAGGAATGGTGTCATCA-3'
	Reverse: 5'- GACTCTACCTGAGGCTTCGAT-3'
GAPDH	Forward: 5'-ATGGGGAAGGTGAAGGTCG-3'
	Reverse: 5'-GGGGTCATTGATGGCAACAATA-3'
p21	Forward: 5'- AGCGATGGAACTTCGACTTTG-3'
	Reverse: 5'- CGAAGTCACCCTCCAGTGGT -3'
MDM2	Forward: 5'- AAATGAATCCCCCCCTTCC -3'
	Reverse: 5'- CACGAAGGGCCCAACATCT-3'
PTEN	Forward: 5'- TTTGAAGACCATAACCCACCAC -3'
	Reverse: 5'- ATTACACCAGTTCGTCCCTTTC-3'
BAX	Forward: 5'- CCCGAGAGGTCTTTTTCCGAG -3'
	Reverse: 5'- CCAGCCCATGATGGTTCTGAT -3'
p21-ChIP	Forward: 5'- GTGGCTCTGATTGGCTTTCTG-3'
	Reverse: 5'- CTCCTACCATCCCCTTCCTC -3'

POX-ChIP	Forward: 5’- GAGGCTTTGAGAAGCCAGTG -3’	
	Reverse: 5’- GAGGCCTCACCCTATATTCCTG-3’	
POX O-site primers	Forward: 5’-GCTAGGTGCAGTCCAGCCTGT-3’	
	Reverse: 5’-CATCTGGATGTATCCGTGC-3’	
POX P-site primers	Forward: 5’-CTACACACCTGTGGGTGTTTC-3’	
	Reverse: 5’-CTAAGGGAACTCAGAGGCTG-3’	
OIP5-AS1	Forward: 5’-TCTGAACTCAGGACTTGGCA-3’	
	Reverse: 5’-ATTCCAAATCATGGAGGTAATG-3’	
***In vivo* Morpholino (Gene tools)**		
Decoys	5’-CTTACTCCGCCATCTTCGCAGCTTC-3’	
	5’-CCCCGGCCCAGAGGCCAGTTT-3’	
**Synthetic oligos (Sangon)**		
POX-130bp (P site in underline)	5’-TACACACCTGTGGGTGTTTCTCGTAAGGTGGGACGAGAGATTTGGAAAAGAAAAAGACACAGAGACAAAGTATAGAGAAAGAAATAAGGGGAACCGGGGAACCAGCGTTCAGCATATGGAGGATCCCGCC-3’	
POX-130bp (O site in underline)	5’-GACCCTGTCTCCACAAAAAAATATTTTTTAAAAATTAGCTAGGTGCAGTCCAGCCTGTAGTCCCAGCTACTTGGGAGGTTGAGGCAGGGGGATCACTTGAGCCCAGGTGTCAGGCCTCTGAGCCCAAGCT-3’	
POX-130bp-mutant1 (mutant1 O site in underline)	5’-GACCCTGTCTCCACAAAAAAATATTTTTTAAAAATTAGCTAGGTGCAGTCCAGCCTGTAGTCCCACGATGAACCCACCTACTCCCACCGGGATCACTTGAGCCCAGGTGTCAGGCCTCTGAGCCCAAGCT-3’	
POX-130bp-mutant2 (mutant2 O site in underline)	5’-GACCCTGTCTCCACAAAAAAATATTTTTTAAAAATTAGCTAGGTGCAGTCCAGCCTGTAGTCCCTCCATAGCCACAGCAATTGCCAGCGGGATCACTTGAGCCCAGGTGTCAGGCCTCTGAGCCCAAGCT-3’	
